# Supplementary material for: Tunable fluorogenic DNA probes drive fast and high-resolution single-molecule fluorescence imaging
Source: Nucleic Acids Res. 2025 Jul 8;53(13):gkaf593. doi: 10.1093/nar/gkaf593 (PMC12235510; doi:10.1093/nar/gkaf593)
Supplement: gkaf593_Supplemental_File [file gkaf593_supplemental_file.docx]

# Supplementary Material

## Determining the Förster Radius:


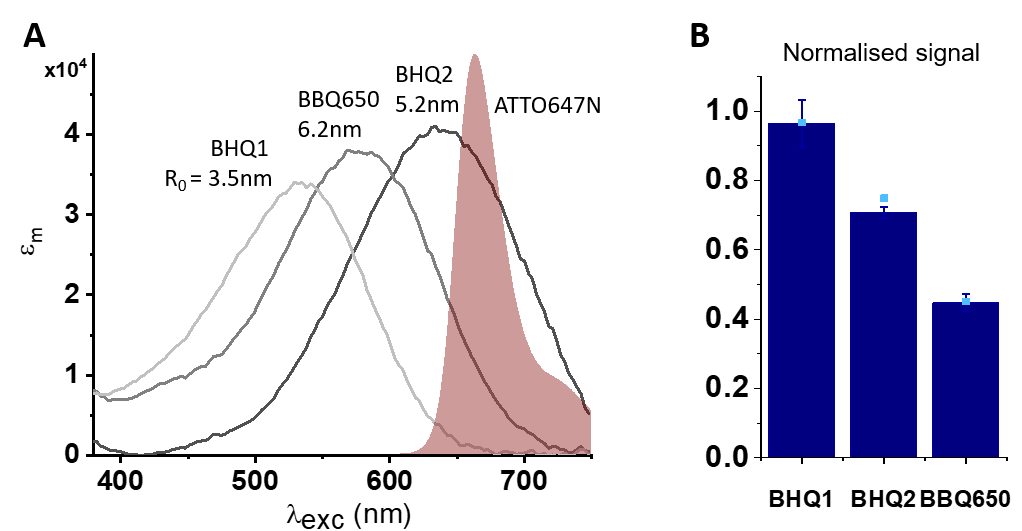


Figure S1: Determination of the Förster Radius. Quencher absorption spectra and the ATTO647N emission spectrum with calculated Förster Radii.

We measured absorption spectra of all Quenchers on the Nanodrop, and used the Fluorophore emission spectra and fluorescence lifetimes provided by the manufacturer.^[1,2]^ The Förster radii were then calculated using the FRET-Calc tool.^[3]^

## Secondary Structural Elements


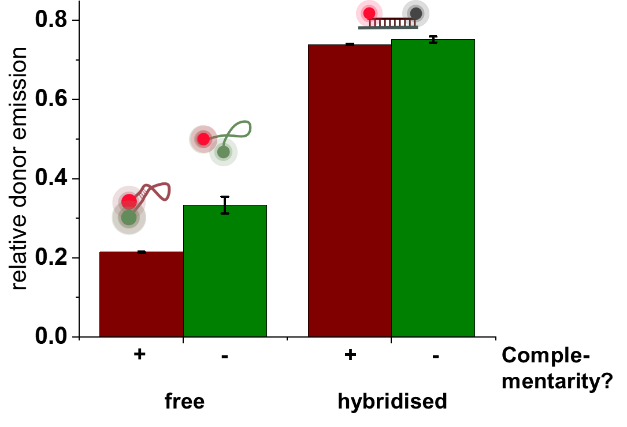


Figure S2: FRET study of self-complementarity effects: A: Cy3 emission levels in single-stranded, free probes. C: Cy3 emission levels in the hybridised, double-stranded state. Mean and SEM of three independent experiments.

To support our hypothesis that self-complementary regions can further compact the probes in the free, single-stranded state, we performed FRET measurements of two different 18nt long constructs labelled with Cy3 (donor) and Cy5 (acceptor, Figure S3). The sequence with 4bp complementarity we studied before showed a relative donor emission of approx. 0.2 (red), whereas for a T-only 18nt probe (green) we measure approx. 0.35 in the ssDNA state. This suggests different average distances between dyes, with the all-T construct being less compact. The difference is lost upon hybridisation to a complementary strand, and the remaining FRET is consistent with the expected distances in an 18nt dsDNA.

Experimental details:

Sequences can be found in Table S1. Oligos were resuspended in buffer (50mM Hepes, 200mM NaCl, 200mM MgCl_2_). Fluorescence emission spectra of 100uL of 100nM probe were measured in a pti fluorimeter (exc: 520nm), with and without 10x excess of complementary DNA. Peak intensities for donor and acceptor emissions were extracted. After background subtraction, the relative donor emission (I_rel,D_) was calculated as follows:

I_rel,D_ = I_D_/(I_D_+I_A_)

Because of changes in quantum yield (QY) of Cy3 upon hybridisation from ssDNA to dsDNA, we have not calculated FRET-efficiencies, but have given the relative donor intensity in the different states, which reflect the different FRET efficiencies. Further, we have not corrected for sequence-specific QY effects with Cy3. Based on the available literature, these may lead to an overestimated donor emission in the probe with secondary structure in the ssDNA state, and so the contrast in the figure is likely underestimating the real distance differences^[4]^.

## Expansion to different spectral regions


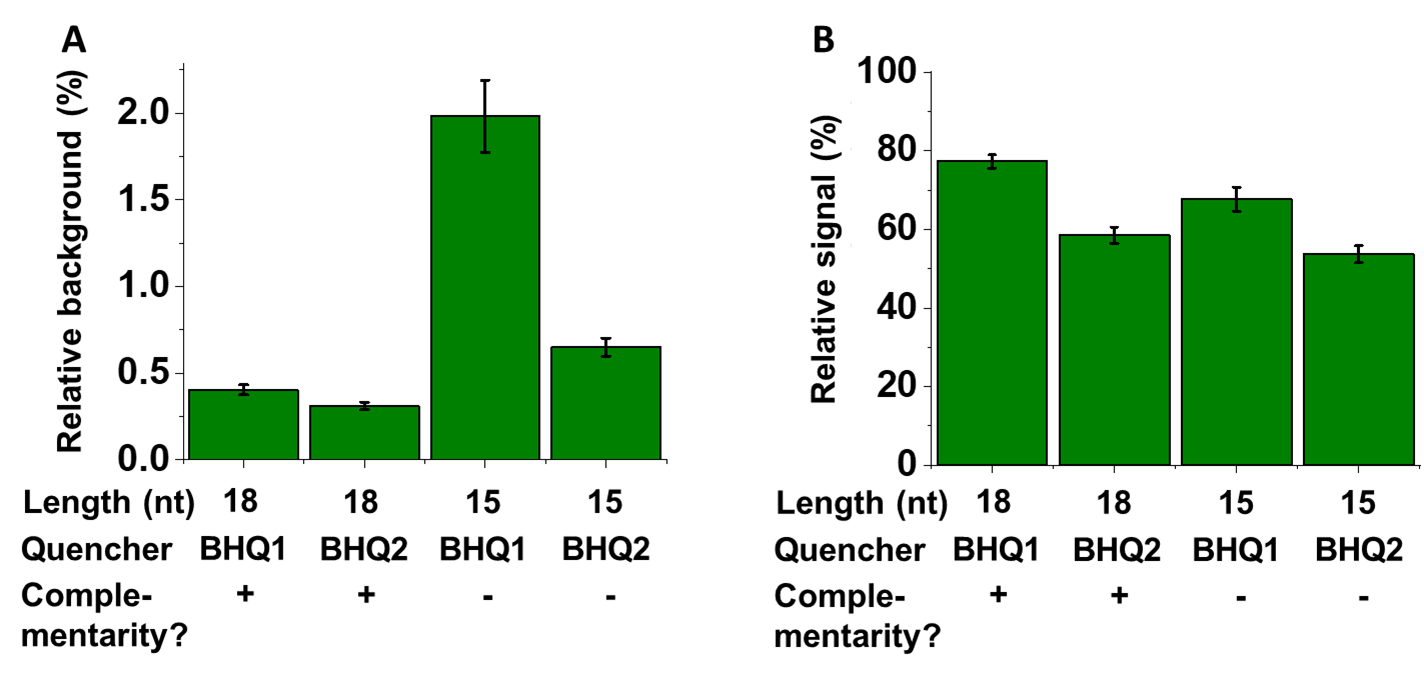


Figure S3: Fluorogenicity in Cy3B-labelled constructs. Relative background (A) and signal (B) levels of constructs of different lengths, quenchers, and sequences. Mean and standard deviation of three independent experiments.

We can extend our findings regarding the effects of the different design parameters to a different spectral region, as demonstrated here using Cy3B as a fluorophore. We tested a 15nt sequence without secondary structural elements and the same 18nt sequence with secondary structure as discussed in the main paper.

The addition of the quencher reduces the background emission in all constructs (down to <5% of the dye-only reference), indicating very efficient quenching. Relative signal levels, however, are all above 50% of the Cy3B-only reference construct. They are lower than observed when Atto647N was paired with the same quenchers at the same probe lengths, which is consistent with the larger spectral overlap between Cy3B emission and the quencher’s absorption spectra, resulting in larger R_0_ values.

Like those constructs with Atto647N, the Cy3B probes exhibit lower background and signal levels when combined with a quencher with a larger R_0_ (BHQ2(6.1nm) vs BHQ1(5.2nm), calculated using the FRET-calc tool^[3]^). The introduction of secondary structural elements allowed the 18nt constructs to overall exhibit lower relative background levels than the shorter 15nt probes without secondary elements.

In Summary, we can recapitulate the main findings of the characterization section with a second, structurally unrelated fluorophore, Cy3B.

Experimental details:

Sequences can be found in Table S1. Relative Signal levels were measured and calculated as outlined in the main body ‘Methods’ section. Relative background levels were extracted from peak intensities of fluorescence spectra of 200nM unbound probes obtained in a pti fluorimeter. All values were normalised to emission levels of a dye-only reference construct.

## DNA-PAINT Specificity

Figure S4: Control experiments for 6nt DNA-PAINT imaging. **A**: Negative control without primary probes (red) shows few localisations compared to the sample with primary probes (cyan). **B:** P1 imager (Cy3B, Cyan) and 6nt imager (ATTO647N, red) colocalise at viral segments.

## Fourier-Ring-Correlation of 6 nt Imager data

Figure S5: Fourier-Ring-Correlation for resolution estimation in the 6nt imager data. Sub-10nm resolution (based on a threshold of 0.143) is achieved after 100-150s of imaging. Mean and SD of three fields-of-view in two independent experiments.

## Sequences of DNAs

| Name | Sequence 5'-> 3' | 5' modification | 3' modification |
| --- | --- | --- | --- |
| **Characterisation** |  |  |  |
| SP_5_A647N_BHQ1 | TTT TT | A647N | BHQ1 |
| SP_6_A647N_BHQ1 | TTT TTT | A647N | BHQ1 |
| SP_8_A647N_BHQ1 | TTT GGT TT | A647N | BHQ1 |
| SP_10_A647N_BHQ1 | TTT GTG GTT T | A647N | BHQ1 |
| SP_12_A647N_BHQ1 | TTT GTT GGT TTT | A647N | BHQ1 |
| SP_15_A647N_BHQ1 | TTT GTT GGT TGG TTT | A647N | BHQ1 |
| SP_20_A647N_BHQ1 | TTT GTT GGT TGG GTT GTT TT | A647N | BHQ1 |
| SP_25_A647N_BHQ1 | TTT GTT GGT TGG GTT GTG TTG GTT T | A647N | BHQ1 |
| SP_6_comp_bio | AAA AAA | biotin |  |
| SP_8_comp_bio | AAA CCA AA | biotin |  |
| SP_10_comp_bio | AAA CCA CAA A | biotin |  |
| SP_12_comp_bio | AAA ACC AAC AAA | biotin |  |
| SP_15_comp_bio | AAA CCA ACC AAC AAA | biotin |  |
| SP_20_comp_bio | AAA ACA ACC CAA CCA ACA AA | biotin |  |
| SP_25_comp_bio | AAA CCA ACA CAA CCC AAC CAA CAA A | biotin |  |
| SP _15_A647N_BHQ2 | TTT GTT GGT TGG TTT | A647N | BHQ2 |
| SP _15_A647N_BBQ650 | TTT GTT GGT TGG TTT | A647N | BBQ650 |
| SP _15_A647N | TTT GTT GGT TGG TTT | A647N |  |
| SP _18_2ndry | GCTGCCTCCCGTAGGAGT | A647N | BHQ1 |
| SP_18_comp_2ndry_bio | ACTCCTACGGGAGGCAGC | biotin |  |
| **DNA-PAINT** |  |  |  |
| P3 | GTAATGAAGA | Cy3B |  |
| 6ntI_A643_BMNQ1 | TGGTGG | Atto 643 | BMNQ1 |
| 6ntI_A647N_BHQ1 | TGGTGG | A647N | BHQ1 |
| P1 | CTAGATGTAT | Cy3B |  |
| comp_R2-6ntI_bio | TTTCCACCA | biotin |  |
| Primary Probes 6nt_PB1 | CCACCACCACCA-R |  | R = comp. PB1 Sequences ^[5]^ |
| Primary Probes P3_NA | TCTTCATTAC-R |  | R= comp. NA Sequences ^[5]^ |
| Primary Probes 6nt_PB1 | TTATACATCTA-R |  | R = comp. PB1 Sequences ^[5]^ |
| **Single-molecule assay** |  |  |  |
| gap_left | CCTCATTCTTCGTCCCA**T**TACCATACA | Cy3B |  |
| gap_right | CGATAATCTGCTGCCTCAGGCTCTTGACTG |  |  |
| seal | TCCACCGT | A643 | BHQ1 |
| bottom_A | CAGTCAAGAGCCTGAGGCAGCAGATTATCGACGGT**A**GATGTATGGTAATGGGACGAAGAATGAGG |  | Biotin |
| bottom_T | CAGTCAAGAGCCTGAGGCAGCAGATTATCGACGGT**T**GATGTATGGTAATGGGACGAAGAATGAGG |  | Biotin |
| bottom_G | CAGTCAAGAGCCTGAGGCAGCAGATTATCGACGGTGGATGTATGGTAATGGGACGAAGAATGAGG |  | Biotin |
| bottom_C | CAGTCAAGAGCCTGAGGCAGCAGATTATCGACGGT**C**GATGTATGGTAATGGGACGAAGAATGAGG |  | Biotin |
| **Supplementary data** |  |  |  |
| 18nt_2ndry_BHQ1 | GCTGCCTCCCGTAGGAGT | Cy3B | BHQ1 |
| 18nt_2ndry_BHQ2 | GCTGCCTCCCGTAGGAGT | Cy3B | BHQ2 |
| 15nt_BHQ1 | aga agt aat gtg gaa | Cy3B | BHQ1 |
| 15nt_BHQ2 | aga agt aat gtg gaa | Cy3B | BHQ2 |
| Cy3B reference | TCC TCC TCC TC | Cy3B |  |
| 18T_Cy3Cy5 | TTTTT TTTTT TTTTT TTT | Cy3 | Cy5 |
| Comp_18T | AAAAA AAAAA AAAAA AAA |  |  |
| 18nt_2ndry_Cy3Cy5 | GCTGCCTCCCGTAGGAGT | Cy3 | Cy5 |
| Comp_18nt_2ndry | ACTCCTACGGGAGGCAGC |  |  |

# References

[1] ATTO-TEC GmbH, “ATTO647N product Information,” **2022**.

[2] ATTO-TEC GmbH, “ATTO643 product information,” **2022**.

[3] L. Benatto, O. Mesquita, J. L. B. Rosa, L. S. Roman, M. Koehler, R. B. Capaz, G. Candiotto, *Comput Phys Commun* **2023**, *287*, 108715.

[4] C. Agbavwe, M. M. Somoza, *PLoS One* **2011**, *6*, e22177.

[5] C. Hepp, Q. Zhao, N. Robb, E. Fodor, A. N. Kapanidis, **2025**, DOI 10.1101/2025.02.11.637713.
